# The Relative Toxity of Cocain and Eucain

**Published:** 1899-10

**Authors:** A. H. Peck

**Affiliations:** Chicago, Professor of Materia Medica, Therapeutics and Special Pathology, in North-western University Dental School, Chicago


### THE DENTAL REGISTER.

VOL. LIII.] OCTOBER, 1899. [No. 10.

### COMMUNICATIONS.

### The Relative Toxity of Cocain and Eucain.

BY A. H. PECK, M.D., D.D.S., CHICAGO.

Professor of Materia Medica, Therapeutics and Special Pathology, in Northwestern University Dental School, Chicago.

Abstract of paper read before the Section of Stomatology, American Medical Association, Columbus, June, 1899.

It is not my intention in this paper to especially refer to the anesthetic properties of cocain, but only its toxic effects relatively with those of eucain, as observed in actual practice and as determined by original experimentation.

Unfortunately cocain is, in its action, one of the most inconstant and unreliable drugs in the whole pharmacopeia. It will always produce anesthesia, if properly used, and also frequently produces poisonous symptoms, oftentimes alarming. If a certain quantity of arsenic or morphia, or almost any other known poison be used under certain circumstances, the resulting symptoms are nearly always the same; we know what to expect. It is unfortunate that the same can not be said of the action of cocain. I have often seen the most alarming symptoms of systemic poisoning result from the use of a certain quantity of cocain, which, in other individuals a like amount under seemingly the same conditions, produces no bad symptoms. There is no other drug in the whole realm of medicine in connection with the action of which individuals vary so much in point of susceptibility. I have seen all the stereotyped symptoms of systemic cocain poisoning result from the use, in a pulp-canal, of a small quantity of a one percent solution, while again I have seen injected into individuals twenty minims of a two percent solution, and this being repeated as high as four times without poisonous symptoms resulting. I firmly believe that cocain produces, in some, local poisoning as well as systemic poisoning, notwithstanding the fact that many disagree with this statement. I have seen from the subcutaneous use of cocain, for the extraction of one root of a tooth, three good teeth lost, with extensive destruction of the alveolar plates of bone, together with extensive sloughing of the soft parts, and all this in the absence of any systemic symptoms. Some, no doubt, will say this is the result of infection by an unclean syringe. But how is it to be proven that this is the case? The work has been done under the most careful antiseptic precautions. I have seen so many cases of local poisoning in varying degrees result from the use of this drug, that I am forced to the belief that it possesses local toxic properties as well as systemic toxic properties.

As in eucain "B," there is much to be said in its favor as compared with cocain. During the past year I have frequently used it in practice for devitalizing pulps, for local applications and by injection, and have as yet observed no evil effects of note. Eucain, however, is not capable of producing the same degree of anesthesia under like circumstances as in cocain. This has been proven beyond the possibility of a doubt by the experiments I have made. As this paper is to deal only with the toxic properties of these drugs, I will not here discuss their anesthetic properties.

Last year, while experimenting extensively with the essential oils, using guinea pigs largely, I did some work with cocain and eucain, enough to demonstrate that there was an interesting field in contrasting the two. Since then I have experimented extensively with these agents, using guinea pigs.

How completely is the statement that cocain varies much in its action demonstrated and proven to be correct by the following experiments: The first one serving to exhibit the poisonous propensities of the drug, and this, too, in the absence of any marked anesthetic effects. A pig, weight eight and one-half ounces, into which was injected twenty minims of a two percent solution of cocain, which amount represents $\frac{3}{20}$ of one grain, after the lapse of eight minutes showed some indications of anethesia, but these were comparatively insignificant. For the next six minutes various symptoms of distress were exhibited, such as a general spasmodic jerky action of all the muscles of the body, accompanied with evidence of pain. At the end of fourteen minutes its hind legs were partially paralyzed and one minute later it fell on its side completely overcome. Its head was firmly drawn back, all the muscles of its body being rigid; this condition would, at short intervals, give way to a distressful spasmodic action, general in its scope, and thus these sets of symptoms continued to alternate for a period of five minutes. During these twenty minutes the animal was not anesthetized to any appreciable extent, responding vigorously to a prick of a needle at any portion of the body. The heart action at first was somewhat depressed, but soon recovered, and thereafter its contractions were strong and rapid, evidently being much stimulated. The respiratory organs, at first slightly stimulated, were very soon depressed, and remained so until recovery set in. After twenty minutes it began to recover and at the end of twenty-five minutes could stand on its feet; however could not walk without falling. At the expiration of forty minutes recovery was far advanced. This experiment shows the toxic action of the drug, with the absence of anesthetic effect, better than any other on my list. While the poisonous properties of this drug are frequently manifested, they are usually accompanied with anesthesia.

[The next experiment cited showed in just as interesting and decided a manner the anesthetic action of the drug to the exclusion of nearly all manifestations of poisonous symptoms, and to the mind of the author, effectually settled the question of inconstancy of the action of cocain.—Ed.]

Let us now study the action of Beta Eucain, under similar circumstances. Twenty minims of a two percent solution of beta eucain, or $\frac{3}{20}$ of one grain, were injected into a pig of eight and one-half ounces weight. At the expiration of thirteen minutes slight evidence of anesthesia—or perhaps better said, the action was that of a mild hypnotic, the animal appearing somewhat drowsy, at the same time all reflexes responding to any interference by way of pricking. The action of the heart and respiratory apparatus was slightly depressed. Three minutes later recovery commenced, and at the expiration of twenty minutes the effects of the drug had largely passed away. Two minutes later another twenty minims, or $\frac{6}{20}$ of one grain in all, were injected. Five minutes later slight evidence of nausea was observed; at nine minutes the hypnotic action was more marked and was accompanied with slight evidence of true anesthesia. Its hind parts were somewhat paralyzed, and the reflexes slightly blunted. The heart action and respiration were more depressed than at first. At eighteen minutes it began to recover and at expiration of twenty-five minutes, or forty-seven minutes from time of first injection, was able to walk about. One minute later, or forty-eight minutes from time of the first injection, a third injection of twenty minims, making $\frac{9}{20}$ of one grain in all, was made. Five minutes later nausea developed and the animal seemed much distressed thereby. The heart action and respiration being at first stimulated, soon became much depressed; after ten minutes twitching of all muscles of the body, spasmodic in character, developed; this condition increased until, at the expiration of eighteen minutes, the animal fell to the table completely exhausted. Its head was firmly drawn back, all the muscles being at high tension; rapid winking of the eyes continued, with gasping for breath and twitching of ears. For the next twenty minutes this condition continued, with violent tetanoid spasms of all muscles following one another in rapid succession, each spasm being accompanied with mournful squealing, which seemed to indicate much distress and pain. At no time were the reflexes, either plantar or cremasteric, lost; neither was there much evidence of general anesthesia; indeed, the entire action seemed to be more that of a paralyzer than of an anesthetizer. The heart action and the respiratory apparatus were much depressed. At times the heart-beat was almost undiscernible, and the animal would gasp distressingly for breath. At the expiration of this twenty minutes, signs of recovery developed and at the expiration of twelve minutes more it could stand on its feet, but could not walk without toppling over. It was now very late, and the animal was placed in its box, and in the morning was found to be none the worse off for its experience. This case is interesting in that it seems to prove conclusively that, at least, three times the quantity of beta eucain is required to produce virtually the same degree of toxicity as is produced by cocain. These results, or this action of the two drugs, as related in this experiment and the first one with cocain, I regard as bearing directly on their toxic properties. Alpha Eucain has proven to be virtually on a par with cocain as to toxic properties. Five-sixths of one grain is the limit as to fatal action with pigs of twenty-five and one-fourth ounces weight.

By way of recapitulation, my experiments lead me to conclude as follows:

1. The action of cocain is inconstant; one never knows whether the symptoms occasioned by like quantities of the drug in animals or individuals, under like circumstances, will be similar or dissimilar.

2. The action of eucain is constant. The symptoms occasioned by the use of like quantities in animals under like circumstances, and so far as my experience has gone, in different individuals also, are the same.

3. That the first action of cocain on the heart is that of a depressant, and on the respiration is that of a mild stimulant; the after effects being, on the heart, that of a decided stimulant, and on the respiration that of a depressant.

4. That the first action of eucain, on both the heart and respiration, is that of a stimulant, the after effects being that of a decided depressant.

5. That cocain causes death in animals by paralyzing the muscles of the respiratory apparatus; the heart's action continuing in a feeble way for a brief period after breathing ceases.

6. That eucain causes death in animals by paralyzing the muscles of the heart and of the respiratory apparatus, they ceasing to operate simultaneously.

7. That eucain in toxic doses nearly always causes nausea, and occasionally vomiting.

8. That cocain is much less nauseating, and scarcely ever causes vomiting.

9. That eucain is decidedly a diuretic, causing renal discharge in a majority of instances in which a toxic dose is used.

10. That cocain is not to any appreciable extent a diuretic, renal discharge having occurred in only one instance in connection with all my experiments.

11. That the pupils of the eyes in nearly all cases of cocain poisoning do not respond to light, and are more or less bulging from their sockets.

12. That the pupils of the eyes in most cases of eucain poisoning do respond feebly to light, and rarely ever bulge from their sockets.

13. That the action of toxic doses of eucain is more like that of a paralyzing, tetanoiding, convulsion-producing agent than it is like an anesthetizing one, the plantar and cremasteric reflexes nearly always responding.

14. That toxic doses of cocain cause general anesthesia, with the other symptoms, in the majority of cases.

15. True tetanus, of all striped muscles of the limbs, and Cheyne-Stokes breathing nearly always occur when eucain is used.

16. That cocain is at least three times more toxic than beta eucain, and that alpha eucain is as toxic as cocain.

17. That boiling does not destroy the efficacy of cocain, but that it does modify it, and that boiling in no degree lessens the efficacy of eucain.

The above conclusions have been formed only after many experiments in connection with each individual point. I have observed in my work many interesting features in connection with the relative worth of these drugs as local anesthetics, but this paper is not to treat of this phase of the work. There is much experimental work yet to be done in this connection, the results of which I shall be pleased to present at some future meeting.

DISCUSSION.

After reading his paper, Dr. Peck produced three guinea pigs and injected them with alpha eucain, beta eucain and hydrochlorate of cocain, in doses intended to show relative toxicity with uniform results.

The first pig weighed thirty-two and one-half ounces, and one grain of hydrochlorate of cocain dissolved in twenty drops of water (a 5 percent solution) was injected hypodermically. In five minutes the pig fell over in convulsions, which became tetonic in eight minutes and one-half. In nine minutes from the time of the injection the pig had ceased to breathe, dying from paralysis of respiration.

The second pig weighed thirty-two ounces. One grain of alpha eucain, dissolved in twenty drops of water (a 5 percent solution) was injected hypodermically as before. In five minutes the heart's action became very weak and respiration short and quick. In seven and one-half minutes from the time of the injection the pig jumped up from the table and fell in violent spasms, which continued for seven and one-half minutes, when the posterior extremities became paralyzed. The anterior extremities never lost their function. The heart and respiration were greatly but not so much depressed as by the cocain. The pig recovered in about an hour.

Pig No. 3 weighed nearly twenty-eight ounces. Into this pig was injected three grains of beta eucain in twenty drops of water. The pig apparently suffered no great inconvenience, except as was made known by grinding his teeth. The respiration and heart's function were slightly depressed, but the pig recovered with spasms or other unhappy symptoms.

### PROFESSIONAL ETHICS.

"Professional Education and Ethics" was the title of a paper read by Dr. A. E. Baldwin, of Chicago, in which he drew attention to the necessity for right character as the great factor in stimulating and engendering right conduct. The paper was illustrated by citations of practitioners, who influenced others for the bad because of selfishness manifested in unjust or questionable business transactions, and also by teachers in colleges who lead impure lives or resort to questionable practices in conducting the business affairs of these institutions. Young men entering the profession will not have high moral ideals, if they do not find these ideals cultivated by respected members of the profession.

In discussing the paper, Dr. Arnold said that the old saying, that a stream could not rise higher than its source, was not true in this case, because there is undoubtedly a greater number of men in the profession to-day possessing higher ideals of morality than ever before, and he believed the standard was even higher than ever before. It would be a great discouragement to many teachers to think that there were no higher attainments to be reached than those they now possessed. The pupil often surpasses the master, and it is well that it is so.

Dr. Noyes agreed with Dr. Arnold that the pupil many times makes higher attainments than his teacher, and yet this ought not to excuse the teacher from doing his full duty, nor be an element of discouragement. When the teacher has put the pupil in the way of acquiring knowledge he has done the very best thing he can for such a pupil, and when he excites him to high moral attainments he certainly has the right to be satisfied with his work, and should be greatly encouraged. In criticising the conduct of individual practitioners for isolated misdemeanors, or institutions for conduct of public benefactions, it is well for us to take into account all the factors that go to make up the case before we draw our conclusions. The gold or amalgam used in a college infirmary is not the only nor even the chief expense, in conducting the clinic in a way to best conserve its purpose and the welfare of its patrons.

Dr. Marshall thought that if we could all fall back on the "golden rule," in its original meaning, we should make higher professional attainments and be recognized by the public for our own true worth.

Dr. Talbot thinks there are degenerate classes in all walks of life. A degenerate teacher could not influence the morals of a young man under his care, except for the bad. Hence it follows that much of the professional discredit can be traced to its source in the school where the *morale* of its teaching force was not what it should be. This condition exists too largely at the present time, but it ought not so to do.

Dr. Bonwill thought there were temptations for the young man in the beginning of his practice. Patients often want the young man to do for them operations which they both know ought not to be done. For avariciousness, vanity, or even unquestioned dishonorable reasons, the struggling young professional man too often degrades himself, because of pressure from an influential patron. The lack of professional courtesy among neighboring practitioners is a grave source of engendering or cultivating unethical practices. We should be more considerate in judging of our neighbor's practices, and indulge sparingly in censorious criticisms of a fellow-practitioner's method of reaching an end which may not meet our approval.

Dr. H. A. Smith did not think our code of ethics obsolete. It was taught in a hundred different ways in every dental college in the land. It was not only taught from the rostrum, but it was honestly and conscientiously lived and magnified in every possible way in our schools to-day as never before. It is true that our good country boys, who come to us without guile, often turn out badly, but can any one lay the charge at the door of the college or its teaching force? Possibly in some cases, yes; but more often the cause can be traced to other causes, such as change of environment, lack of experience or training, or of natural grit, which will enable a man to stand amidst the surroundings in which he of necessity finds himself placed. Colleges can not be criticised for working for people who are able to pay for a practitioner's services, nor can they be charged with dishonorable practices when they charge twenty-five cents for five cents' worth of gutta percha or amalgam.

Dr. Baldwin, in closing, thought it wrong in principle for a college clinic to advertise work free or at most only to charge for cost of material, and then charge for cleaning or extracting teeth, or five times as much as the material used had cost. The reflection will be caught up by the student and built into his own life and practice. It is one thing to preach the code of ethics to your students, but it is a far better thing to practice it where they can see it, and value it for what it does and is.

### SYPHILITIC INFECTION.

Dr. W. L. Baum, of Chicago, read a paper on "Syphilitic Infection from Dental Instruments," citing several cases which had come under his own observation.

Dr. Noyes called attention to the fact that it was not always easy to trace the source of such accidents. He knew of a case of inoculation from a public cigar-cutter. But could readily see how it was quite probable that many times there were dangerous conditions in the mouth which the dentist failed to recognize.

Dr. Marshall had never seen a case of inoculation which could be traced to a dentist, but had known of several cases coming from the hands of the rhinologist. Dentists take care of their instruments as well as it is possible, but it may be that insufficient care of the hands and fingers will account for possible inoculations, because of the great difficulty in thoroughly sterilizing the hands without injury to the skin.

Dr. Talbot cited a case of a patient having a specific disease, and who underwent a long and unsuccessful treatment for pyorrhea alveolaris at the hands of a dental specialist. The question naturally arises, how many other patients were endangered by this specialist not recognizing the condition of his patient?

Dr. Crawford did not think syphilis was often transmitted by means of dental operation. The fluids of the mouth are a great protection to the tissues of the mouth, either in furnishing unsuitable media for germ growth or by washing them into the stomach where the gastric juices are more capable of destroying them. We should be able to diagnose syphilitic conditions and so avoid possibilities of inoculation. A physician should never send a syphilitic patient to a dentist without previous systemic treatment, and even then the dentist should be warned of the condition of the patient. A physician who does not do so is willfully negligent of a very important duty.

Dr. Arnold thought the paper timely, as it sounded the alarm, and would warn both the practitioner of medicine and dentistry that they each had an important duty in such cases in bringing each other into helpful and harmonious action. The chances of inoculation from dental was confined to a few operations and instruments which it would be well if dentists could habitually care for in all cases.

Dr. Chamberlain, a physician, related the case of a dentist who extracted several teeth for a patient whom he knew to be a syphilitic, and without cleansing his forceps and an elevator which he used, he rolled the instruments up in a leather case with the blood still on them.

Dr. Baum said dentists can not shirk the responsibility of giving proper attention to cleanliness and the use of local antiseptic applications in these cases because they do not recognize the existence of the disease. They should be able to diagnose it. The oral cavity is no more immune than other tissues, a lesion is necessary for inoculation, and a scratch is more dangerous than a bleeding wound. There is no competent and practicable sterilizing agent for all cases. It is impracticable to completely sterilize the mouth, but there are abundant methods which will make it possible to sterilize the instruments used at least. Have seen many cases of accidental inoculation on the fingers of surgeons and physicians, but have observed none on the hands of dentists.

Dr. J. S. Marshall, of Chicago, read a very interesting paper on "Infectious Ulcerative Stomatitis."

Dr. Crawford highly commended the paper. He was particularly interested to learn that la grippe was considered an active cause of this trouble. He approved the treatment indicated by Dr. Marshall, but would suggest also the application as a disinfectant escharotic to the ulcerated patches, as the eschar would serve to protect the sensitive tissues.

Dr. G. V. I. Brown could not endorse irritant or escharotic treatment, because of liability to unnecessary destruction of tissue or exaggerated inflammation. He preferred simple disinfection with such non-poisonous agents as peroxide of hydrogen.

Dr. H. A. Smith cited a case of sloughing of the mucous membrane of the entire lower jaw.

Dr. Talbot said that by many this disease is thought to be a form of, or intimately associated with, pyorrhea alveolaris, but it is more probably due to inanition or faulty metabolism. Suggested thorough cleansing with plenty of water, containing some mild disinfectant, such as listerine.

Dr. Heise could not agree with the statement that benign tumors never become malignant.

Dr. Bonwill said he was interested in the paper, as it promised to call the attention of dental practitioners to these cases, and would cause them to study these conditions so that they might prescribe for them and cure them as readily as the physician.

Dr. Noyes cited a case of a young lady for whom he was regulating the teeth, who presented herself on one occasion with a serious case of stomatitis. The mucous membrane of the nose and throat were also badly inflamed. At the time the young lady's father was suffering from erysipelas. At the time I feared infection from possible contagion.

Dr. Marshall stated that he would use nitrate of silver in apthous stomatitis or in stomatitis with isolated patches. But in general and diffused ulcerative stomatitis, he would prefer the zinc chloride, because of its astringent as well as disinfectant and antiseptic action. This condition is not often due to the presence of a specific systemic disease, as it develops too rapidly. It does not become malignant, as develops from the superficial tissues. Neither can it be associated with pyorrhea alveolaris. A pyorrhea develops very slowly and ulcerative stomatitis with great rapidity.

Dr. G. V. I. Brown showed a large photograph of a young man who had been burned on the face and neck while a lad. The cicatricial tissue had greatly disfigured the young man, and in a measure made him helpless. By several operations the case was materially bettered, and the patient presented a very much improved appearance. There was a resection of the lower incisor and cuspid teeth with a good union and very satisfactory result. Dr. Brown explained the details of the operation and was generally congratulated on the success of the operation.

Dr. Bonwill showed how such operations could be readily made by using the dental surgical engine. Exhibiting his improved dental engine, which he hoped soon to place on the market.

Several papers were read as a symposium, on pyorrhea alveolaris and diseases of the peridental membrane, and discussed together. These papers were by Dr. Bonwill, on its cure by methods to restore normal articulation; by Dr. Fletcher, on "Neuralgia due to Periosteal Necrosis;" by Dr. Carpenter, on "The Treatment of Persistent Pyorrhea Alveolaris;" by Dr. Noyes, on "The Histological Structure of the Peridental Membrane," and Dr. Talbot, on "Interstitial Gingivitis."

All of these papers will be printed, with the exception of Dr. Talbot's, which appears as a chapter in his new book, just issued. He gave the result of a long series of investigations on dogs, to determine the prevalence of pyorrhea alveolaris in these animals. He has had a large number of dogs from the dog pounds of Chicago and Milwaukee examined, and finds that 25 percent of dogs less than four years old have pyorrhea; 80 percent of those from five to ten years have it, and 95 percent of those from twelve to fourteen years have it. Dr. Talbot has found there is more than one method of degenerative absorption. Besides the usual osteoclast absorbent method, he recognizes a senile degeneration, or osteomalacia, and a third inflammatory process through the Haversian canals, accompanied with destruction of the arterial coats.

A general and somewhat brief discussion was had of the papers.

Dr Marshall endorsed, unqualifiedly, Dr. Bonwill's statement that after operating for pyorrhea, the wounds should not be continually irritated by prolonged instrumentation. The operation should be completed, if possible, at the first sitting, and then allowed to heal, using only medication as indicated to encourage healing and prevent infection. He would object to Dr. Fletcher's application of the term caries to the peridental membrane; preferred suppuration or gangrene. He would also object to the term, serumal deposits, but believed abscesses may be formed in the interstitial structures from the blood. In regard to the epithelial structures, shown in the slides of Dr. Noyes, he thought they could be accounted for as remnants of embryological development.

Dr. Fletcher could not consider the structures shown by Dr. Noyes normal, but would regard them as anomalous. Nature does not build such tissues elsewhere in the body, and why here? The cells are not shaped like epithelial cells, and do not react to the stains from the other cells. He thought they were mesoblastic in origin. Dr. Fletcher thought the destructive onslaught on the peridental membrane as of bacterial origin, and the periosteum broke down without suppuration, because it could not resist the onslaughts of the micro-organisms. The destruction was similar in kind to a chemical abrasion.

Dr. Heise could not fully endorse Dr. Fletcher's interpretation of the dissolving process which takes place in the peridental structures. In Dr. Carpenter's suggestion of extirpating pulps and excising roots of teeth, he saw a dangerous practice if indulged to any considerable extent. While it might assist in eradicating the disease, it would certainly impair the usefulness of the teeth and shorten their existence.

Dr. Noyes said that if the structures shown in his slides were mesoblastic, as Dr. Fletcher suggested, they could not be glandular. They are located in a mesoblastic structure, but he believed they were epithelial in character. They react to all the characteristics of epithelial structures.

Dr. Brown thought pyorrhea was of local origin, often caused, at least influenced, by accidental conditions or circumstances. He was not prepared to say that mal-occlusion of the teeth would entirely account for it, but no doubt it would be instrumental in producing certain characteristic absorptions. He thought operative interferences to correct mal-occlusions might be of great value.

Dr. Carpenter thought the two great factors in the cure of pyorrhea were cleanliness and rest, and yet a certain amount of intelligent medication will be necessary in many cases. He would recommend as to the pockets as a stimulating antiseptic, the following:

℞ Carbonate of creosote.
Oil of cassia - - - - - aa ʒj
Iatrol q. s., add for a paste.

Dr. Heise recommended a similar application, a paste composed of iodoform in lactic acid to proper consistence.

Dr. Bonwill, in discussing Dr. Hill's paper, objected to the use of calomel and jalap as revulsive cathartics. They produced excessive drastic catharsis, and would result in more harm than good. He preferred to treat peridental congestion with a bistoury, producing considerable bleeding, by rapidly stabbing into the gum, over the affected tooth. He would not send his patient to a medical man for systemic treatment, but would resort to local operative measures.

Dr. Brown could not agree with Dr. Bonwill. Patients are often saved much suffering by subjecting them to a season of systemic treatment preparatory to dental operations. Has had very satisfactory consultations with medical men. Physicians can and do relieve pain promptly by early and proper application of systemic anodynes.

